# Supplementary material for: Vitamin D Supplementation and Its Impact on Mortality and Cardiovascular Outcomes: Systematic Review and Meta-Analysis of 80 Randomized Clinical Trials
Source: Nutrients. 2023 Apr 7;15(8):1810. doi: 10.3390/nu15081810 (PMC10146299; doi:10.3390/nu15081810)
Supplement: Supplementary file 1 [file nutrients-15-01810-s001.zip › nutrients-2301270-supplementary.pdf]

Vitamin D Supplementation and Its Impact on Mortality and Cardiovascular Outcomes: Systematic Review and Meta-analysis of 80 Randomized Clinical Trials

Supplementary Materials

Table S1. Terms and search strategies.

• **Search terms:** "Vitamin D", "Calcitriol", "Calcifediol", "Cholecalciferol", "25-Hydroxyvitamin D 2", "Ergocalciferols", “calcium and vitamin D”, “cardiovascular disease”, “cardiovascular risk factors”, “coronary heart disease”, “myocardial infarction”, “stroke”, “heart failure” and “morbidity and/or mortality”.<br>• **Medical Subject Headings (MeSH):** *mortality; calcitriol [*therapeutic use]; cause of death; cholecalciferol [*therapeutic use]; dietary supplements; ergocalciferol [*therapeutic use]; hydroxycholecalciferols [*therapeutic use]; randomized controlled trials as topic; vitamins [*therapeutic use]<br>• **AND:** vitamin D; humans; adult: 19+ years;<br>• **NOT:** pregnancy; lactation; frail elderly; neoplasms; coronavirus; renal dialysis; renal insufficiency; acquired immunodeficiency syndrome; hospitalization; inpatient.<br>• **Abbreviations**: '$': stands for any character; '?': substitutes one or no character; adj: adjacent (i.e. number of words within range of search term); exp: exploded MeSH; MeSH: medical subject heading (MEDLINE medical index term); ab: abstract; ot: other term; pt: publication type; sh: MeSH; ti: title; tw: text word.<br>• **Filters:** Full text, Randomized Controlled Trial, Humans, English, Adult: 19+ years, Exclude preprints, from 1983 – 2022.
• **EMBASE**<br>1. exp ergocalciferol/ or exp vitamin D/<br>2. exp colecalciferol/<br>3. exp dihydrotachysterol/<br>4. exp 25 hydroxyvitamin D/<br>5. exp hydroxycolecalciferol/<br>6. (vitamin* D? or vitamin*D?).tw,ot.<br>7. (cholecalciferol* or colecalciferol* or calcifediol* or calcitriol* or dihydrotachysterol* or hydroxyvitamin* d?).tw,ot.<br>8. exp alfacalcidol/<br>9. (alfacalcidol* or alphacalcidol*).tw,ot.<br>10. or/1-9<br>11. exp mortality/<br>12. (mortality or mortaliti*).tw,ot.<br>13. 11 or 12<br>14. exp prevention/<br>15. prevent*.tw,ot.<br>16. exp neoplasm/<br>17. or/14-16

18. randomized controlled trial/
19. double blind procedure/
20. single blind procedure/
21. exp randomization/
22. exp controlled clinical trial/
23. or/18-22
24. exp meta analysis/
25. (metaanaly$ or meta analy$ or meta?analy$).ab,ti,ot.
26. ((review$ or search$) adj10 (literature$ or medical database$ or medline or pubmed or embase or cochrane or cinahl or psycinfo or psyclit or healthstar or biosis or current content$ or systematic$)).ab,ti,ot.
27. exp Literature/
28. exp Biomedical Technology Assessment/
29. hta.tw,ot.
30. (health technology adj6 assessment$).tw,ot.
31. or/24-30
32. (comment or editorial or historical-article).pt.
33. 31 not 32
34. 23 or 33
35. 10 and 13 and 34
36. 10 and 17 and 34
37. 35 or 36
38. limit 37 to human

- **PubMed**

1. exp vitamin D/
2. exp cholecalciferol/
3. exp ergocalciferol/ or exp dihydrotachysterol/ or exp 25-hydroxyvitamin d 2/
4. exp Hydroxycholecalciferols/
5. vitamin D?.tw,ot.
6. (cholecalciferol$ or calcifediol$ or calcitriol$ or dihydrotachysterol$ or hydroxyvitamin$ d?).tw,ot.
7. (alfacalcidol$ or alphacalcidol$ or colecalciferol$).tw,ot.
8. or/1-7
9. exp Mortality/
10. mortality.tw,ot.
11. mortaliti$.tw,ot.
12. or/9-11
13. exp Primary Prevention/
14. (prevention$ or prevent$).tw,ot.
15. exp Neoplasm/
16. (cancer$ or neoplasm$ or tumo?r$).tw,ot.
17. or/13-16
18. exp Randomized Controlled Trials as topic/
19. Randomized Controlled Trial.pt.
20. exp Controlled Clinical Trials as topic/
21. Controlled Clinical Trial.pt.
22. exp Random Allocation/

23. exp Double-Blind Method/
24. exp Single-Blind Method/
25. or/18-24
26. exp "Review Literature as topic"/
27. exp Technology Assessment, Biomedical/
28. exp Meta-analysis as topic/
29. Meta-analysis.pt.
30. hta.tw,ot.
31. (health technology adj6 assessment$).tw,ot.
32. (meta analy$ or metaanaly$ or meta?analy$).tw,ot.
33. ((review$ or search$) adj10 (literature$ or medical database$ or medline or pubmed or embase or cochrane or cinahl or psycinfo or psyclit or healthstar or biosis or current content$ or systemat$)).tw,ot.
34. or/26-33
35. 25 or 34
36. 8 and 17 and 35
37. 8 and 12 and 35
38 36 or 37
39. limit 38 to animals
40. limit 38 to humans
41. 39 not 40
42. 38 not 41

- **The Cochrane Library**

1. MeSH descriptor Vitamin D explode all trees
2. MeSH descriptor Cholecalciferol explode all trees
3. MeSH descriptor Ergocalciferols explode all trees
4. MeSH descriptor Dihydrotachysterol explode all trees
5. MeSH descriptor 25-hydroxyvitamin D 2 explode all trees
6. MeSH descriptor Hydroxycholecalciferols explode all trees
7. ((vitamin* in All Text and d in All Text and 2 in All Text) or (vitamin* in All Text and d2 in All Text))
8. (cholecalciferol* in All Text or calciferol* in All Text or calcitriol* in All Text or dihydrotachysterol* in All Text or (hydroxyvitamin* in All Text and d* in All Text))
9. (alfacalcidol* in All Text or alphacalcidol* in All Text or cholecalciferol* in All Text)
10. (#1 or #2 or #3 or #4 or #5 or #6 or #7 or #8 or #9)
11. MeSH descriptor Mortality explode all trees
12. (mortality in All Text or mortaliti* in All Text)
13. (#11 or #12)
14. MeSH descriptor Primary Prevention explode all trees
15. prevent* in All Text
16. MeSH descriptor Neoplasms explode all trees
17. (#14 or #15 or #16)
18. (#10 and #13)
19. (#10 and #17)
20. (#18 or #19)

Table S2. Characteristics of good-quality RCTs [22-56].

**Study, authors. publication year**	**Location**	**Intervention vitamin D3 *** (IU)	**Intervention vitamin D2*** (IU)	**Intervention 1α(OH)D *** (mcg)	**Intervention eldecalcitol*** (mcg)	**Intervention calcium *** (mg)	**Control: placebo or calcium *** (mg)	**Treatment median** (years)	**Follow-up median** (years)	**Intervention** (No)	**Control** (No)
Lips et al. 1996	Netherlands	400						3.50	3.50	1291	1287
Meyer et al. 2002	Norway	400						2.00	2.00	569	575
BDS, Trivedi et al. 2003	UK	822						5.00	5.00	1345	1341
Flicker et al. 2005	Australia		1429			600	600	2.00	2.00	313	312
Bolton et al. 2007	UK	400				1000		2.00	2.00	62	61
Lyons et al. 2007	UK		822					3.00	3.00	1725	1715
WHI, Hsia et al. 2007	USA	400				1000		7.00	7.00	18176	18106
Prince et al. 2008	Australia		1000			1000	1000	1.00	1.00	151	151
Zhu et al. 2008	Australia		1000			1200		5.00	5.00	39	81
Vital D, Sanders et al. 2010	Australia	1370						2.96	2.96	1131	1127
Lehouck et al. 2012	Belgium	3571						1.00	1.00	91	91
VIDARIS, Murdoch et al. 2012	New Zealand	10989						1.50	1.50	161	161
Davidson et al. 2013	USA	12695						1.00	1.00	56	53
VitDISH, Witham et al. 2013	UK	3333						1.00	1.00	80	79
RECORD, Ford et al. 2014	UK	800				1000	1000	3.00	3.00	2649	2643
Baron et al. 2015	USA	1000				37% 1200	37% 1200	3.80	3.80	1130	1129
Hansen et al. 2015	USA	800-3333						1.00	1.00	154	76
Uusi-Rasi et al. 2015	Finland	800						2.00	2.00	204	205
ViDiCO, Martineau et al. 2015	UK	2000						1.00	1.00	122	118

VIDEO, Arden et al. 2016	UK	800						3.00	3.00	237	237
Brisson et al. 2017	Canada	1000-3000						1.00	1.00	306	99
Lappe et al. 2017	USA	2000				1500		4.00	4.00	1156	1147
BEST-D, Hin et al. 2017	UK	2000-4000						1.00	1.00	204	101
ViDA, Scragg et al. 2017	New Zealand	3333						3.30	3.30	2558	2550
Nahas et al. 2018	Brazil	1000						0.75	0.75	80	80
ViDOS, Smith et al. 2018	USA	400-4800				600	600	1.00	1.00	172	22
Shea et al. 2019	USA	858						1.00	1.00	49	51
VITAL, Manson et al. 2019	USA	2000				50% 1g EPA-DHA	50% 1g EPA-DHA	5.30	5.30	12927	12944
Jeong et al. 2020	Korea	800				1000		1.00	1.00	445	512
Lukenda et al. 2020	Croatia	1000						1.00	1.00	201	110
Yang et al. 2020	China	800						1.00	1.00	93	90
DO-HEALTH, Bischoff et al. 2020	5 European countries	2000				50% 1g EPA-DHA	50% 1g EPA-DHA	2.99	2.99	1076	1081
VIDAL, Rake et al. 2020	UK	3333						1.00	1.00	802	813
DPVD, Kawahara et al. 2022	Japan				0.75			3.00	3.00	630	626
D-Health, Neale et al. 2022	Australia	2000						5.00	5.00	10661	10649
FIND, Virtanen et al. 2022	Finland	1600-3200				1350-1413	1344	5.00	5.00	1665	830

RCTs: randomized clinical trials; *: daily dose; SD: standard deviation; IQR: interquartile range; No: participants number; EPA-DHA: eicosapentaenoic acid-docosahexaenoic acid.

Table S3. Characteristics of fair-quality RCTs [57-90]

**Study, authors. publication year**	**Location**	**Intervention vitamin D3*** (IU)	**Intervention vitamin D2*** (IU)	**Intervention 1α(OH)D*** (mcg)	**Intervention 1,25(OH)₂D*** (mcg)	**Intervention calcium*** (mg)	**Control: placebo or calcium*** (mg)	**Treatment median** (years)	**Follow-up median** (years)	**Intervention** (No)	**Control** (No)
Ott & Chesnut. 1989	USA				0.43	1000	1000	2.00	2.00	43	43
Dawson et al. 1991	USA	400				377	377	1.00	1.00	138	138
Chapuy et al. 1992	France	800				1200		1.50	1.50	1634	1636
Chen et al. 1997	Japan			0.75		150	150	1.00	1.00	25	25
Dawson et al. 1997	USA	700				500		3.00	3.00	202	187
Baeksgaard et al. 1998	Denmark	560				1000		2.00	2.00	80	80
Krieg et al. 1999	Switzerland	880				1000		2.00	2.00	124	124
KORFPS, Komulainen et al. 1999	Finland	300				500		5.00	5.00	116	116
STOP IT, Gallagher et al. 2001	USA				0.50			3.00	5.00	123	123
Decalyos II, Chapuy et al. 2002	France	800				1200		2.00	2.00	389	194
Cooper et al. 2003	Australia		1429			1000	1000	2.00	2.00	93	94
Larsen et al. 2004	Denmark	400				1000		3.50	3.50	4957	4648
Aloia et al. 2005	USA	800-2000				1350	1350	3.00	3.00	104	104
Brazier et al. 2005	France	800				1000		1.00	1.00	95	97
Porthouse et al. 2005	UK	800				1000		2.08	2.08	1321	1993
VIP, Campbell et al. 2005	New Zealand	1667-3333						1.00	1.00	195	196
Lappe et al. 2007	USA	1000				1500	1500	4.00	4.00	446	733
Smith et al. 2007	UK		822					3.00	3.00	4727	4713
Pfeifer et al. 2009	Austria Germany	800				1000	1000	1.00	1.00	121	121

Zitterman et al. 2009	Germany	3332				1.00	1.00	100	100
OSTPRE-FPS, Kärkkäinen et al. 2010	Finland	800		1000		3.00	3.00	290	313
TIDE, Punthakee et al. 2012	International	1000				5.50	5.50	607	614
VICtORy, Wood et al. 2012	UK	400-1000				1.00	1.00	203	102
ViDA, Mason et al. 2014	USA	2000				1.00	1.00	109	109
VITADAS, Gallagher et al. 2014	USA	400-2400				1.00	1.00	160	38
DIVA, Barengolts et al. 2015	USA		7143	400	400	1.00	1.00	103	102
Jin et al. 2016	Australia	1667				2.00	2.00	209	204
Jorde et al. 2016	Norway	2857				5.00	5.00	256	255
VINDICATE, Witte et al. 2016	UK	4000				1.00	1.00	114	109
EVIDENCE, Moreira et al. 2017	Canada	4000				0.46	0.46	35	36
EVITA, Zittermann et al. 2017	Germany	4000				3.00	3.00	199	201
D2d, Pittas et al. 2019	USA	4000				2.50	2.50	1211	1212
PODA, Aloia et al. 2019	USA	3490		1200	1200	3.00	3.00	130	130
CALGB, Charlamb et al. 2021	USA	2000				1.00	1.00	142	148
Hu et al. 2023	China	800				2.50	2.50	135	135

RCTs: randomized clinical trials; *: daily dose; SD: standard deviation; IQR: interquartile range; No: participants number.

Table S4. Characteristics of low-quality RCTs [91-101]

**Study, authors. publication year**	**Location**	**Intervention vitamin D3*** (UI)	**Intervention vitamin D2*** (mg)	**Intervention calcium*** (mg)	**Control: placebo or calcium*** (mg)	**Treatment median** (years)	**Follow-up median** (years)	**Intervention** (No)	**Control** (No)
Inkovaara et al. 1983	Finland	1000		1200 (50%)	1200 (50%)	0.75	1.00	91	84
Meier et al. 2004	Germany	500		500		0.50	1.00	30	25
NONOF, Harwood et al. 2004	UK	800	822	1000		1.00	1.00	113	37
Moschonis & Manios 2006	Greece	300		1200		1.00	1.00	42	70
Daly & Nowson 2009	Australia	800		1000		2.00	3.50	85	82
Nieves et al. 2012	USA	1000		1000	1000	2.00	2.00	64	63
Toss & Magnusson 2012	Sweden	1600		1000	1000	1.00	1.00	28	28
Kuchay et al. 2015	India	2466				1.00	1.00	69	68
Sinha-Hikim et al. 2015	USA	12186				1.00	1.00	40	40
PREVENT-WIN, Bhatt et al. 2020	India	200-8571		1000	1000	1.50	1.50	61	60
Vahdat et al. 2022	Iran	1000				1.00	1.00	55	54

RCTs: randomized clinical trials; *: daily dose; SD: standard deviation; IQR: interquartile range; No: participants number.

Table S5. Baseline characteristics of participants from good-quality RCTs [22-56]

Study, authors. publication year	Participants (No)	Age Mean ± SD Median (IQR) (years)	Female (%)	Current smoking (%)	HTN (%)	Prediabetes (%)	DM (%)	High cholesterol or lowering medication (%)
Lips et al. 1996	2 578	80 ± 6	74.3					
Meyer et al. 2002	1 144	84.8	75.9					
BDS, Trivedi et al. 2003	2686	74.8 ± 4.6	24.2	4.2				
Flicker et al. 2005	625	83.4 ± 8.3	95.0					
Bolton et al. 2007	123	68	100.0					
Lyons et al. 2007	3 440	84 ± 7.5	76.3					
WHI, Hsia et al. 2007	36 282	62.4 ± 6.9	100.0	7.7	33.3		5.8	12.2
Prince et al. 2008	302	77.2 ± 4.6	100.0					
Zhu et al. 2008	120	75 (70-80)	100.0					
Vital D, Sanders et al. 2010	2 258	76.0 (73-80)	100.0					
Lehouck et al. 2012	182	68	20.3					
VIDARIS, Murdoch et al. 2012	322	47.5 ± 10.0	74.8	5.3			1.2	
Davidson et al. 2013	109	52.4 ± 7.5	81.6			100	0	
VitDISH, Witham et al. 2013	159	76.8 ± 4.7	48.4		100		14	
RECORD, Ford et al. 2014	5 292	77.5 ± 6	84.7	11.5			7.9	
Baron et al. 2015	2259	58.3	37.0	11.6				
Hansen et al. 2015	230	61.0 ± 6.0	100.0					
Uusi-Rasi et al. 2015	409	74.2 ± 3.0	100.0	19.3	44.7		9	
ViDiCO, Martineau et al. 2015	240	64.7 ± 8.1	40.0	40.8				
VIDEO, Arden et al. 2016	474	64 ± 8	61.0					
Brisson et al. 2017	405	57.8 ± 5.5	100.0					
Lappe et al. 2017	2 303	65.2 ± 7.0	100.0	6.1				
BEST-D, Hin et al. 2017	305	72 ± 6	49.0	12	39		9	27.5
ViDA, Scragg et al. 2017	5 108	65.9 ± 8.3	41.9	6.3	36.9		9.9	
Nahas et al. 2018	160	59.1 ± 6.7	23.7					
ViDOS, Smith et al. 2018	194	66.7 ± 7.2	100.0	10.4				
Shea et al. 2019	100	69.6 ± 6.9	36.0					62.0
VITAL, Manson et al. 2019	25 871	67.1 ± 7.1	50.6	7.2	49.8		13.7	37.5
Jeong et al. 2020	957	60.9 ± 11.9	64.4					
Lukenda et al. 2020	311	65.0 (21.5-84.0)	42.8		52.7		55	47.9

Yang et al. 2020	183	66.9 ± 5.7	55.2	7.1	36.6		14.8	
DO-HEALTH, Bischoff et al. 2020	2 157	75.0 ± 4.5	61.7	5.8	49.6			
VIDAL, Rake et al. 2020	1 615	65-84	46.9					
DPVD, Kawahara et al. 2022	1 256	61.3 ± 8.9	45.5			100	0	
D-Health, Neale et al. 2022	21 310	69.3 ± 5.5	44.3	4.2	41.6		8.3	32.2
FIND, Virtanen et al. 2022	2 495	68.2	43.3	35.7	42.3		9.0	29.0

RCTs: randomized clinical trials; No: number; HTN: arterial hypertension; DM:

Table S6. Baseline characteristics of participants from fair-quality RCTs [57-90]

**Study, authors. publication year**	**Participants** (No)	**Female** (%)	**Age Mean ± SD Median (IQR)** (years)	**Current smoking** (%)	**HTN** (%)	**Prediabetes** (%)	**DM** (%)	**High cholesterol or lowering medication** (%)
Ott & Chesnut. 1989	86	100.0	67.5					
Dawson et al. 1991	276	100.0	61.7 ± 0.5	7.4				
Chapuy et al. 1992	3 270	100.0	84 ± 6					
Chen et al. 1997	50	50.0	52.3 ± 0.7					
Dawson et al. 1997	389	55.0	71 ± 4	5.7				
Baeksgaard et al. 1998	160	100.0	62.5 (58-67)					
Krieg et al. 1999	248	100.0	84.5 ± 7.5					
KORFPS, Komulainen et al. 1999	232	100.0	52.7 (47-56)				2.2	
STOP IT, Gallagher et al. 2001	246	100.0	71.5					
Decalyos II, Chapuy et al. 2002	583	100.0	85.2 ± 7.1					
Cooper et al. 2003	187	100.0	56.3 ± 4.5					
Larsen et al. 2004	9 605	60.1	74 (66-103)					
Aloia et al. 2005	208	100.0	60.5 ± 6.3	6.7				
Brazier et al. 2005	192	100.0	74.6 ± 6.9					
Porthouse et al. 2005	3314	100.0	76.8					
VIP, Campbell et al. 2005	391	68.0	83.6 ± 4.8					
Lappe et al. 2007	1 179	100.0	66.7 ± 7.3					
Smith et al. 2007	9440	53.9	79.1 (76.9-82.7)					
Pfeifer et al. 2009	242	89.0	76.5 ± 4.0					
Zitterman et al. 2009	200	69.3	48.1 ± 10.2					
OSTPRE-FPS, Kärkkäinen et al. 2010	603	100.0	67. 4 ± 2.0	4.8	53.9			
TIDE, Punthakee et al. 2012	1 221	40.9	66.6 ± 6.5	12.4	87.8		100	76.6
VICtORy, Wood et al. 2012	305	100.0	63.8 ± 2.2					
ViDA, Mason et al. 2014	218	100.0	59.6 ± 5.1					
VITADAS, Gallagher et al. 2014	198	100.0	36.7 ± 5.9	18				
DIVA, Barengolts et al. 2015	205	0.0	59.0 ± 6.0	58.5	13.2		45.9	
Jin et al. 2016	413	50.0	63.2 ± 7.1					

Jorde et al. 2016	511	38.6	62.1 ± 8.6	20.7	47.0	100.0	0	26.6
VINDICATE, Witte et al. 2016	223	20.9	68.7 ± 13.1				22.7	
EVIDENCE, Moreira et al. 2017	71	53.5	47.3 ± 14.3			49.3	11.3	
EVITA, Zittermann et al. 2017	400	17.0	55 (18-79)		30.0		24.3	54.5
D2d, Pittas et al. 2019	2 423	44.8	60.0 ± 9.9					
PODA, Aloia et al. 2019	260	100.0	68.2 ± 4.9	4.6				
CALGB, Charlamb et al. 2021	290	100.0	42.6 ± 6.3					
Hu et al. 2023	270	73.6	66.1 ± 8.6				100.0	

RCTs: randomized clinical trials; No: number; HTN: arterial hypertension; DM: diabetes mellitus.

Table S7. Baseline characteristics of participants from low-quality RCTs [91-101]

**Study, authors. publication year**	**Participants** (No)	**Female** (%)	**Age Mean ± SD Median (IQR)** (years)	**Current smoking** (%)	**HTN** (%)	**Prediabetes** (%)	**DM** (%)
Inkovaara et al. 1983	175	81.7	79.3 ± 6.6				
Meier et al. 2004	55	65	56.5 ± 11.1	13.7			
NONOF, Harwood et al. 2004	150	100.0	81.2 (67-92)				
Moschonis & Manios 2006	112	100.0	60.3				
Daly & Nowson 2009	167	0.0	61.3 ± 7.7	4.9			20.2
Nieves et al. 2012	127	100.0	61.8 ± 8.1				
Toss & Magnusson 2012	56	71.4	70.0 ± 8.0				
Kuchay et al. 2015	137	50.0	47 ± 11			100	0
Sinha-Hikim et al. 2015	80	70.0	52.0 ± 7.2			100	0
PREVENT-WIN, Bhatt et al. 2020	121	100.0	(20-60)	3.3	36.4		0
Vahdat et al. 2022	109	100.0	37.9 ± 6.5				

RCTs: randomized clinical trials; *: daily dose; SD: standard deviation; IQR: interquartile range; No: number; HTN: arterial hypertension; DM: diabetes mellitus.

Table S8. RCTs included according to outcomes analysis

ACM	CVM	Non-CVM	MI	Stroke	HF	MACE	MACE+
Lips et al. 1996							
Meyer et al. 2002							
BDS, Trivedi et al. 2003	BDS, Trivedi et al. 2003	BDS, Trivedi et al. 2003	BDS, Trivedi et al. 2003	BDS, Trivedi et al. 2003	BDS, Trivedi et al. 2003		
Flicker et al. 2005							
Bolton et al. 2007	Bolton et al. 2007	Bolton et al. 2007					
Lyons et al. 2007							
WHI, Hsia et al. 2007	WHI, Hsia et al. 2007	WHI, Hsia et al. 2007	WHI, Hsia et al. 2007	WHI, Hsia et al. 2007	WHI, Hsia et al. 2007	WHI, Hsia et al. 2007	
Prince et al. 2008			Prince et al. 2008	Prince et al. 2008		Prince et al. 2008	
Zhu et al. 2008							
OSTPRE-FPS, Kärkkäinen et al. 2010							
Vital D, Sanders et al. 2010	Vital D, Sanders et al. 2010	Vital D, Sanders et al. 2010				Vital D, Sanders et al. 2010	
Lehouck et al. 2012							
VIDARIS, Murdoch et al. 2012	VIDARIS, Murdoch et al. 2012	VIDARIS, Murdoch et al. 2012					
Davidson et al. 2013	Davidson et al. 2013	Davidson et al. 2013					
VitDISH, Witham et al. 2013							
RECORD, Ford et al. 2014	RECORD, Ford et al. 2014	RECORD, Ford et al. 2014	RECORD, Ford et al. 2014	RECORD, Ford et al. 2014	RECORD, Ford et al. 2014	RECORD, Ford et al. 2014	
Baron et al. 2015			Baron et al. 2015	Baron et al. 2015		Baron et al. 2015	

Hansen et al. 2015	Hansen et al. 2015	Hansen et al. 2015					
Uusi-Rasi et al. 2015							
ViDiCO, Martineau et al. 2015							
VIDEO, Arden et al. 2016							
Brisson et al. 2017	Brisson et al. 2017	Brisson et al. 2017					
Lappe et al. 2017							
BEST-D, Hin et al. 2017							
ViDA, Scragg et al. 2017	ViDA, Scragg et al. 2017	ViDA, Scragg et al. 2017	ViDA, Scragg et al. 2017	ViDA, Scragg et al. 2017	ViDA, Scragg et al. 2017	ViDA, Scragg et al. 2017	
Nahas et al. 2018	Nahas et al. 2018	Nahas et al. 2018					
ViDOS, Smith et al. 2018	ViDOS, Smith et al. 2018	ViDOS, Smith et al. 2018					
Shea et al. 2019	Shea et al. 2019	Shea et al. 2019					
VITAL, Manson et al. 2019	VITAL, Manson et al. 2019	VITAL, Manson et al. 2019	VITAL, Manson et al. 2019	VITAL, Manson et al. 2019		VITAL, Manson et al. 2019	VITAL, Manson et al. 2019
Jeong et al. 2020	Jeong et al. 2020	Jeong et al. 2020					
Lukenda et al. 2020	Lukenda et al. 2020	Lukenda et al. 2020					
Yang et al. 2020	Yang et al. 2020	Yang et al. 2020					
DO-HEALTH, Bischoff et al. 2020							
VIDAL, Rake et al. 2020	VIDAL, Rake et al. 2020	VIDAL, Rake et al. 2020					
DPVD, Kawahara et al. 2022							

D-Health, Neale et al. 2022	D-Health, Neale et al. 2022	D-Health, Neale et al. 2022					
FIND, Virtanen et al. 2022	FIND, Virtanen et al. 2022	FIND, Virtanen et al. 2022	FIND, Virtanen et al. 2022	FIND, Virtanen et al. 2022		FIND, Virtanen et al. 2022	FIND, Virtanen et al. 2022
Ott & Chesnut. 1989							
Dawson et al. 1991	Dawson et al. 1991	Dawson et al. 1991					
Chapuy et al. 1992							
Chen et al. 1997	Chen et al. 1997	Chen et al. 1997					
Dawson et al. 1997							
Baeksgaard et al. 1998							
Krieg et al. 1999							
KORFPS, Komulainen et al. 1999							
STOP IT, Gallagher et al. 2001			STOP IT, Gallagher et al. 2001	STOP IT, Gallagher et al. 2001		STOP IT, Gallagher et al. 2001	
Decalyos II, Chapuy et al. 2002							
Cooper et al. 2003							
Larsen et al. 2004							
Aloia et al. 2005							
Brazier et al. 2005	Brazier et al. 2005	Brazier et al. 2005	Brazier et al. 2005	Brazier et al. 2005		Brazier et al. 2005	
Porthouse et al. 2005							

VIP, Campbell et al. 2005							
Lappe et al. 2007							
Smith et al. 2007							
Pfeifer et al. 2009	Pfeifer et al. 2009	Pfeifer et al. 2009					
Zitterman et al. 2009	Zitterman et al. 2009	Zitterman et al. 2009					
OSTPRE-FPS, Kärkkäinen et al. 2010							
TIDE, Punthakee et al. 2012	TIDE, Punthakee et al. 2012	TIDE, Punthakee et al. 2012	TIDE, Punthakee et al. 2012	TIDE, Punthakee et al. 2012	TIDE, Punthakee et al. 2012	TIDE, Punthakee et al. 2012	TIDE, Punthakee et al. 2012
VICtORy, Wood et al. 2012	VICtORy, Wood et al. 2012	VICtORy, Wood et al. 2012					
ViDA, Mason et al. 2014	ViDA, Mason et al. 2014	ViDA, Mason et al. 2014					
VITADAS, Gallagher et al. 2014	VITADAS, Gallagher et al. 2014	VITADAS, Gallagher et al. 2014					
DIVA, Barengolts et al. 2015	DIVA, Barengolts et al. 2015	DIVA, Barengolts et al. 2015					
Jin et al. 2016			Jin et al. 2016				
Jorde et al. 2016			Jorde et al. 2016	Jorde et al. 2016		Jorde et al. 2016	
VINDICATE, Witte et al. 2016							
EVIDENCE, Moreira et al. 2017	EVIDENCE, Moreira et al. 2017	EVIDENCE, Moreira et al. 2017					
EVITA, Zittermann et al. 2017	EVITA, Zittermann et al. 2017	EVITA, Zittermann et al. 2017					
D2d, Pittas et al. 2019							
PODA, Aloia et al. 2019							

CALGB, Charlamb et al. 2021						
Hu et al. 2023	Hu et al. 2023	Hu et al. 2023				
Inkovaara et al. 1983	Inkovaara et al. 1983	Inkovaara et al. 1983	Inkovaara et al. 1983	Inkovaara et al. 1983		Inkovaara et al. 1983
Meier et al. 2004						
NONOF, Harwood et al. 2004						
Moschonis & Manios 2006	Moschonis & Manios 2006	Moschonis & Manios 2006				
Daly & Nowson 2009						
Nieves et al. 2012	Nieves et al. 2012	Nieves et al. 2012				
Toss & Magnusson 2012	Toss & Magnusson 2012	Toss & Magnusson 2012				
Kuchay et al. 2015	Kuchay et al. 2015	Kuchay et al. 2015				
Sinha-Hikim et al. 2015	Sinha-Hikim et al. 2015	Sinha-Hikim et al. 2015				
PREVENT-WIN, Bhatt et al. 2020	Ebadi et al. 2021	Ebadi et al. 2021				
Vahdat et al. 2022	Vahdat et al. 2022	Vahdat et al. 2022				

ACM: all-cause mortality; CVM: cardiovascular mortality; Non-CVM: non-cardiovascular mortality; MI: myocardial infarction; HF: heart failure; MACE: major adverse cardiovascular events; MACE+: MACE or coronary revascularization procedures (percutaneous coronary intervention or coronary artery bypass grafting).
